# Add-On *Bifidobacterium Bifidum* Supplement in Children with Attention-Deficit/Hyperactivity Disorder: A 12-Week Randomized Double-Blind Placebo-Controlled Clinical Trial

**DOI:** 10.3390/nu16142260

**Published:** 2024-07-13

**Authors:** Liang-Jen Wang, Ching-Shu Tsai, Wen-Jiun Chou, Ho-Chang Kuo, Ying-Hsien Huang, Sheng-Yu Lee, Hong-Ying Dai, Chia-Yu Yang, Chia-Jung Li, Yao-Tsung Yeh

**Affiliations:** 1 Department of Child and Adolescent Psychiatry, Kaohsiung Chang Gung Memorial Hospital, Chang Gung University College of Medicine, Kaohsiung 83301, Taiwan; wangliangjen@gmail.com (L.-J.W.); jingshutsai@yahoo.com.tw (C.-S.T.); wjchouoe2@gmail.com (W.-J.C.); parissweettoast@gmail.com (C.-J.L.); 2 Institute for Translational Research in Biomedicine, Kaohsiung Chang Gung Memorial Hospital, Chang Gung University College of Medicine, Kaohsiung 83301, Taiwan; 3 Department of Pediatrics, Kaohsiung Chang Gung Memorial Hospital, Chang Gung University College of Medicine, Kaohsiung 83301, Taiwan; erickuo48@yahoo.com.tw (H.-C.K.); yhhuang123@yahoo.com.tw (Y.-H.H.); 4 Kawasaki Disease Center, Kaohsiung Chang Gung Memorial Hospital, Kaohsiung 83301, Taiwan; 5 Department of Psychiatry, Kaohsiung Veterans General Hospital, Kaohsiung 813, Taiwan; shirleylee.ncku@gmail.com; 6 Department of Psychiatry, College of Medicine, Kaohsiung Medical University, Kaohsiung 807378, Taiwan; 7 Aging and Disease Prevention Research Center, Fooyin University, Kaohsiung 83102, Taiwan; maxhungying7000@gmail.com; 8 Department of Microbiology and Immunology/Molecular Medicine Research Center, Chang Gung University, Taoyuan 333, Taiwan; chiayu-yang@mail.cgu.edu.tw

**Keywords:** ADHD, *Bifidobacterium*, gut–brain axis, microbiome, probiotic, psychobiotics

## Abstract

We conducted a 12-week randomized double-blind placebo-controlled clinical trial to investigate the potential impact of *Bifidobacterium bifidum* (*Bf-688*) supplementation on attention-deficit/hyperactivity disorder (ADHD). Children with ADHD who were already receiving a stable dose of methylphenidate (MPH) treatment were enrolled and were randomly assigned to two groups: one receiving add-on *Bf-688* (daily bacterial count of 5 × 10^9^ CFUs) (*n* = 51) and the other receiving a placebo (*n* = 51). All participants underwent assessments using Conners’ Continuous Performance Test (CPT) and Conners’ Continuous Auditory Test of Attention (CATA). Additionally, fecal samples were collected at the beginning of the trial (week 0) and at the endpoint (week 12). Remarkably, the group receiving *Bf-688* supplementation, but not the placebo group, exhibited significant improvements in omission errors in CPT as well as Hit reaction time in both CPT and CATA. Gut microbiome analysis revealed a significant increase in the *Firmicutes* to *Bacteroidetes* ratio (*F/B* ratio) only in the *Bf-688* group. Furthermore, we identified significant negative correlations between N-Glycan biosynthesis and Hit reaction time in both CPT and CATA. Our results demonstrate that the probiotic *Bf-688* supplement can enhance neuropsychological performance in children with ADHD, possibly by altering the composition of the gut microbiota, ultimately leading to reduced N-Glycan biosynthesis.

## 1. Introduction

Attention-deficit/hyperactivity disorder (ADHD) stands out as one of the most prevalent neurodevelopmental disorders, affecting roughly 8% of school-age children worldwide [1,2]. Recently, there has been a growing focus on the complex and bidirectional communication pathways between the gut microbiota and the central nervous system [3,4], often referred to as the “gut–brain axis” [5]. This axis is implicated in various physical aspects, including dietary patterns, neurotransmitters, the endocrine system, immunological processes, and gut permeability [6,7,8,9]. The gut microbiome has the potential to influence early human brain development and may play a role in the pathophysiology of ADHD [10,11,12,13]. Psychobiotics, the modern term for a type of probiotic, appear to work through dopamine, epinephrine, noradrenaline, and 5-hydroxytryptamine [14]. Manipulating the microbiome through probiotic supplements in individuals with ADHD holds promise for uncovering novel therapeutic approaches [15,16,17,18,19,20,21,22].

The *Lactobacillus* family of probiotics, *Lactobacillus rhamnosus* (*LGG*), is the most frequently studied and is known to modulate the relationship between the neural-immune system, neuroendocrine system, and the central nervous system [23,24,25,26,27]. In addition, research from Sweden reported that Synbiotic 2000 could alleviate autism symptoms and enhance emotional regulation in children [28], potentially through the reduction in intestinal and vascular inflammation and the elevation of short-chain fatty acid (SCFA) levels [29]. Not only *Lactobacillus* strains but also members of the *Bifidobacterium* genus show promise in the treatment of neurodevelopmental disorders in children, such as ADHD [30]. The *Bifidobacterium* genus is linked to the synthesis of dopamine precursors and is considered one of the biomarkers associated with ADHD [31,32]. In a prior animal study, supplementing with *Bifidobacterium* breve during early life appeared to help reduce hyperactivity in adolescent rats with low birthweight [33]. Additionally, a 10-week randomized control trial involving micronutrient supplementation altered the abundance of *Bifidobacterium* and had the potential to influence ADHD behavior in children [34]. Among the *Bifidobacterial* communities, the *Bifidobacterium bifidum* (*B. bifidum*) species stands out as one of the predominant taxa [35]. *B. bifidum G9-1* (*BBG9-1*) has been found to ameliorate dysbiosis, leading to an increase in organic acids and an enhancement of neurotransmission, including dopamine [36]. Furthermore, our previous open-label study also revealed that supplementation with a specific strain of *B. bifidum*, the probiotic *Bf-688*, was associated with an improvement in symptoms related to inattention and hyperactivity/impulsiveness [37].

Pharmacological treatments have demonstrated their effectiveness in addressing the symptoms of ADHD and enhancing various functional aspects, including quality of life, academic performance, and reducing accident rates [38]. Methylphenidate (MPH), acting as a dopamine and norepinephrine reuptake inhibitor, stands out as the most commonly prescribed psychostimulant for ADHD [39]. Nonetheless, MPH is known to bring about common adverse effects in children and adolescents, including reduced appetite, weight loss, and abdominal discomfort [40,41]. A meta-analysis revealed the prevalence of MPH side effects such as sleep disturbances (17.9%), headaches (14.4%), abdominal pain (10.7%), and reduced appetite (31.1%) [42]. Heat-inactivated *B. bifidum MIMBb75* (*SYN-HI-001*) has been shown to significantly alleviate the symptoms of irritable bowel syndrome [43]. Furthermore, our prior open-label study revealed that supplementation with the probiotic *Bf-688* was associated with weight gain in drug-naïve children with ADHD [37]. Hence, we posited that the inclusion of *Bf-688* as an adjunct treatment could yield advantages in alleviating gastrointestinal symptoms and promoting weight gain in individuals with ADHD who are also receiving pharmacotherapy. Moreover, we anticipated that augmenting the treatment with *Bf-688* might prove beneficial in addressing ADHD symptoms and rectifying microbiota imbalances.

Consequently, we conducted a randomized double-blind placebo-controlled clinical trial with the primary objective of assessing the impact of *Bf-688* add-on therapy on ADHD clinical presentation, neuropsychological performance, and body weight progression in children undergoing pharmacotherapy for ADHD. The secondary outcome focused on the potential alteration of gut microbiome composition during the clinical trial.

## 2. Materials and Methods

### 2.1. Study Participants

This research protocol received approval from the Institutional Review Board (IRB) at Chang Gung Memorial Hospital in Taiwan (Approval No. 202100880A3). The trial has been registered with ClinicalTrials.gov (NCT04958460). We recruited eligible patients with ADHD from the Outpatient Department of Child Psychiatry at Chang Gung Children’s Hospital in Taiwan. Before participants were enrolled in this study, we provided a detailed explanation of the study protocols to both the participants and their parents or legal guardians. Written informed consent was obtained from both the child and the parent/guardian once they agreed to participate in the study.

The eligibility criteria for patients with ADHD were as follows: (a) a confirmed clinical diagnosis of ADHD by an experienced child psychiatrist, determined through structured interviews based on the Diagnostic and Statistical Manual of Mental Disorders (DSM–5) criteria [44,45]; (b) age between 6 and 12; and (c) ongoing pharmacotherapy for ADHD at a stable dosage for a minimum of four weeks.

Conversely, individuals meeting any of the following criteria were excluded from this study: (a) patients with a history of significant neuropsychiatric conditions, including intellectual disabilities, autism spectrum disorder, bipolar disorders, major depressive disorders, psychotic disorders, or substance use disorders; (b) patients with major underlying physical illnesses, such as genetic or endocrine disorders, severe head trauma, or gastrointestinal disorders; and (c) patients who followed a vegetarian diet or were currently taking probiotics or antibiotics.

### 2.2. Allocation

We conducted power calculations to determine the sample size for the randomized controlled trial (RCT) [46]. We utilized an online sample size calculator available at https://clincalc.com/stats/samplesize.aspx (accessed on 28 June 2021). Based on the findings from our preliminary open-label study [37], the estimated sample sizes required for this double-blind placebo-controlled randomized clinical trial were determined to be *n*  =  45 in each group.

In this clinical trial, which followed a randomized double-blind placebo-controlled design, a total of 107 children between the ages of 6 and 12 years, meeting the criteria for an ADHD diagnosis, were recruited. The assignment of patients was conducted using a computer-generated random number, with participants being assigned in a 1:1 ratio to either the *Bf-688* group (*n* = 54) or the placebo group (*n* = 53).

Individuals in the *Bf-688* group were administered *Bf-688* for a duration of 12 weeks, with one sachet taken both in the morning and evening, resulting in a daily bacterial count of 5 × 10^9^ CFUs. Conversely, participants in the placebo group were provided with placebo packets consisting of maltodextrin and corn starch, also taken twice daily for a period of 12 weeks.

Patients were instructed to adhere to their prescribed MPH treatment consistently, maintaining a stable dosage. Compliance with the medication regimen was verified at each visit by assessing patient reports provided by their parents and examining any remaining medication. The use of other concurrent medications was prohibited. Additionally, all participants were instructed not to make any alterations to their dietary habits or lifestyle during the study.

### 2.3. Study Procedures

**Figure 1:**
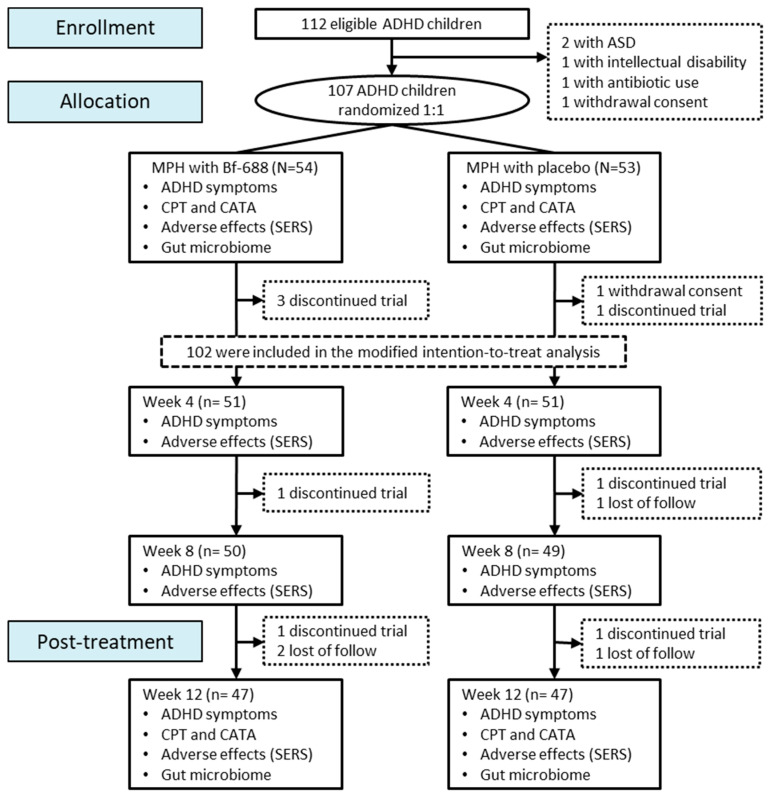
CONSORT diagram for study procedure and flowchart. During the second visit at week 4, three individuals from the *Bf-688* group discontinued their participation in the trial. This included two participants who found the taste of the probiotics unacceptable and one who ceased taking ADHD medication. In the placebo group, one participant withdrew from the trial due to an aversion to the taste of the placebo, and another participant withdrew their consent.

The patients followed the aforementioned prescription regimen consistently for a duration of 12 consecutive weeks. The study protocol and the follow-up schedule are depicted in Figure 1. At four different time points, specifically at the baseline, the 4th week, the 8th week, and the 12th week, the following assessments and measurements were conducted: recording of the children’s height, body weight, and body mass index (BMI). The assessment of ADHD symptoms was conducted using two established tools: the Swanson, Nolan, and Pelham Rating Scale (SNAP-IV) [47,48] and the ADHD rating scale (ADHD-RS) [49]. Parents of the children were asked to complete the SNAP-IV parent form [47,48] as well as Barkley’s Side Effects Rating Scale (SERS) [50], and the clinical symptoms were assessed by the researcher using the ADHD-RS [49]. In order to explore any potential benefits of *Bf-688* concerning gastrointestinal (GI) symptoms, we separately analyzed the cumulative scores for stomachaches and loss of appetite [51].

Additionally, at both the baseline (week 0) and the conclusion (week 12) of the study, a child psychologist administered the Conners’ Continuous Performance Test (Conners CPT) [52] and Conners’ Continuous Auditory Test of Attention (CATA) [53] to evaluate the patients’ visual and auditory attention abilities, respectively. Previous research has demonstrated that the neuropsychological tests CPT3 and CATA provide objective information pertaining to ADHD cases [54,55]. The specific measures employed in the analyses encompass detectability (d’), omissions, commissions, and Hit reaction time (RT).

### 2.4. Gut Microbiome Analysis

All participants were instructed to provide fecal samples both at the study’s outset (week 0) and at its conclusion (week 12). The extraction of bacterial DNA from these fecal samples was carried out using the QIAamp Fast DNA Stool Mini Kit, manufactured by Qiagen in Hilden, Germany, albeit with some adjustments to the standard protocol.

In brief, the stool sample underwent a centrifugation step at 13,200 rpm for 10 min to eliminate the storage buffer, followed by lysis utilizing InhibitEX buffer by Qiagen in Hilden, Germany. After homogenization, proteinase K and ethanol were introduced to process the resulting supernatant. Subsequently, the supernatant underwent a wash step using a QIAamp spin column, with the final elution being carried out using elution buffer. The concentration of the extracted DNA was assessed using a NanoDrop 2000 (Thermo Fisher Scientific, Waltham, MA, USA), and a subsequent 10× dilution was performed using elution buffer.

### 2.5. Next Generation Sequencing (NGS) Analysis

The gut microbiome library was constructed by targeting the standard V3-V4 region of the 16S rRNA gene. PCR amplification was performed using KAPA HiFi hotstart readymix from Roche in the United States, and subsequent purification was accomplished using AMPure XP magnetic beads manufactured by Beckman Coulter (Brea, CA, USA).

To ensure the quality and quantity of the PCR product, an assessment was conducted using a Fragment Analyzer, which is a product from Advanced Analytical in the United States. Additionally, quantification was carried out using a Qubit 3.0 Fluorometer (Thermo Fisher, Waltham, MA, USA). Following these steps, the library underwent sequencing on a MiSeq platform provided by Illumina in the United States, utilizing paired-end reads with dimensions of 2 × 301 nt for each sample.

### 2.6. Data Analysis

Statistical analysis was carried out using IBM’s SPSS version 22 software (Armonk, NY, USA). Continuous data were presented as either mean ± standard error of the mean or standard deviation, and comparisons were made using an independent *t*-test. For categorical data, percentages were used, and comparisons were performed using the chi-squared test.

When analyzing the longitudinal data, we adhered to the modified intention-to-treat (ITT) principle [56]. Specifically, participants who were randomized to receive treatment and had taken at least one dose of probiotics or placebo (as evidenced by their presence at week 4) were included in the analysis. To address missing data at week 8 and week 12, we employed the last observation carried forward method (LOCF). To assess the extent of change in various outcome measures over the course, we employed a mixed-model analysis of variance (ANOVA). This analysis allowed us to examine within-group differences (referred to as the “time effect”), between-group differences (comparing the *Bf-688* group to the placebo group), and potential interactions between time and groups. The homogeneity of variances and covariances was examined using Levene’s test to ensure that the distribution of results was close to normal [57,58]. Statistical significance was indicated by *p*  <  0.05.

We conducted sequence data quality control and constructed the feature table using QIIME 2 version 2023.2 (https://qiime2.org, accessed on 26 May 2023) [59] in conjunction with the DADA2 pipeline [60] for correction. Alpha diversity, which characterizes the diversity within a specific area or ecosystem, was assessed using metrics such as the Shannon and Chao-1 indices. Chao-1 was employed to gauge community richness, with higher values indicating greater abundance. The Shannon index was used to measure bacterial diversity, with higher values signifying a more diverse community. For beta diversity analysis, we utilized MicrobiomeAnalyst (version 2.0) [61]. Taxonomic compositions of the Amplicon Sequence Variants (ASVs) were mapped based on the Greengenes 13_8 99% Operational Taxonomic Units (OTUs) as reference sequences [62].

All data presented in this study are expressed as means ± standard deviation. The plots were generated using the Python packages seaborn and matplotlib [63]. To investigate differentially abundant bacterial taxa between groups, we performed bidirectional hierarchical clustering analysis based on different grouping information using the R package heatmap. Functional analysis, specifically Phylogenetic Investigation of Communities by Reconstruction of Unobserved States (PICRUSt), was conducted using the Galaxy/Hutlab website (https://huttenhower.sph.harvard.edu/galaxy/, accessed on 3 July 2023).

Statistical significance was defined as a *p*-value less than 0.05. Further adjustments for statistical significance were made using the False Discovery Rate (FDR). The FDR correction involved determining the smallest Benjamini–Hochberg adjusted *p*-value when applying unpaired *t*-tests with Welch’s correction. An FDR-adjusted *p*-value, also referred to as a q-value, of 0.05 indicates that 5% of significant tests may result in false positives.

## 3. Results

### 3.1. Clinical Outcome Analysis

A total of 107 children diagnosed with ADHD who met the screening criteria were recruited and then divided into two groups: the *Bf-688* group, comprising 54 participants, and the placebo group, with 53 participants. All 107 participants successfully completed the initial baseline assessment, as illustrated in Figure 1. In total, we proceeded with a modified ITT analysis involving 51 subjects in both the *Bf-688* group (average age: 9.1 years, 82.4% boys) and the placebo group (average age: 9.1 years, 88.2% boys). The characteristics of these 102 participants are detailed in Table 1. Importantly, at baseline, no significant differences were observed across various factors, including demographic data, ADHD subtypes, comorbidities, MPH doses, side effects, clinical symptoms, and neuropsychological test results.

**Figure 2:**
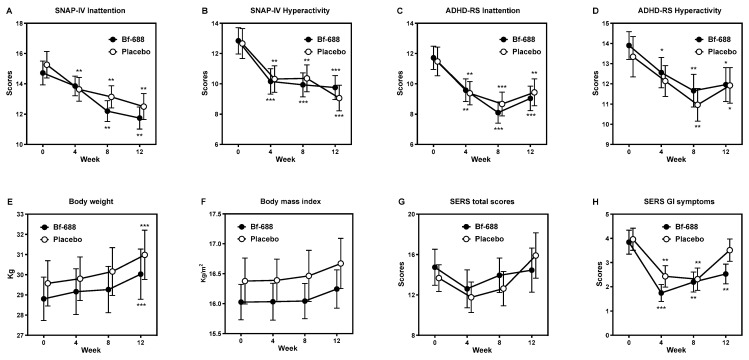
ADHD symptoms, body weight, BMI, and adverse effects of children with ADHD pharmacotherapy over the course of a 12-week double-blind placebo-controlled trial. (**A**) SNAP-IV Inattention symptoms; (**B**) SNAP-IV Hyperactivity symptoms; (**C**) ADHD-RS Inattention symptoms; (**D**) ADHD-RS Hyperactivity symptoms; (**E**) Body weight; (**F**) Body mass index; (**G**) SERS total scores; and (**H**) SERS GI symptoms. Bf-688, *Bifidobacterium Bifidum* plus ADHD medication for 12 weeks; Placebo, placebo plus ADHD medication for 12 weeks; SNAP-IV, the Swanson, Nolan, and Pelham Rating Scale; ADHD-RS, ADHD rating scale; SERS, Barkley’s Side Effects Rating Scale; SERS GI (gastrointestinal) symptoms, severity of stomachache, and loss of appetite. * *p* < 0.05, ** *p* < 0.01, *** *p* < 0.001 compared with baseline data.

As illustrated in Figure 2, both the *Bf-688* group and the placebo group exhibited similar patterns in their SNAP-IV scores, ADHD-RS scores, body weight, BMI, and SERS total scores over the course of the 12-week study period. Specifically, scores related to inattention and hyperactivity on the SNAP-IV and ADHD-RS scales registered significant reductions in both groups during this period.

Moreover, in both the *Bf-688* and placebo groups, the children’s body weight increased by the end of the 12-week period compared to their baseline measurements. Notably, BMI and SERS total scores displayed no significant changes throughout the study duration when compared to their initial values. Interestingly, GI symptoms assessed using the SERS scale significantly decreased in the *Bf-688* group between week 4 and the endpoint at week 12. However, in the placebo group, GI symptoms saw a notable reduction at both week 4 and week 8, but experienced an upward trend, returning to their baseline levels by the study’s endpoint at week 12.

**Figure 3:**
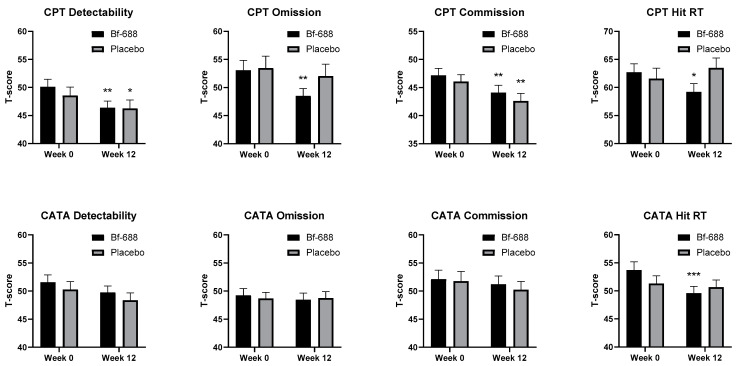
Patients’ visual and auditory attention of children with ADHD pharmacotherapy at the baseline (week 0) and the endpoint (week 12). CPT, Conners’ Continuous Performance Test; CATA, Conners’ Continuous Auditory Test of Attention; Hit reaction time (RT). * *p* < 0.05, ** *p* < 0.01, *** *p* < 0.001 compared with baseline data.

Figure 3 illustrates the levels of visual attention (CPT) and auditory attention (CATA) in children receiving ADHD pharmacotherapy in both groups at two time points: week 0 and week 12. After 12 weeks, both groups displayed significant improvements in commissions and detectability (d’). However, the *Bf-688* group showed significant enhancements in omission errors of CPT and Hit RT in both CPT and CATA, while no such improvements were observed in the placebo group.

### 3.2. Gut Microbiome Analysis

To explore the influence of the gut–brain axis, fecal samples were collected from patients before and after 12 weeks of consistent administration of the prescribed treatment. In total, 76 patients successfully provided fecal samples, comprising 37 individuals from the *Bf-688* group and 39 from the placebo group. We utilized 16S rRNA gene sequencing to track changes in the gut microbial community.

**Figure 4:**
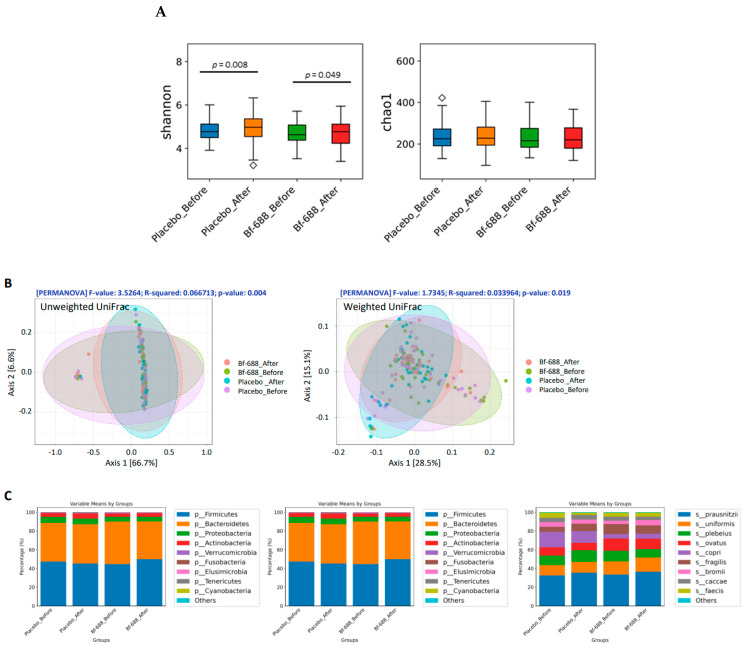
Analysis of abundant bacteria (**A**) Shannon and Chao-1 analyses revealed significant differences in both the placebo and *Bf-688* groups before and after probiotic supplementation. (**B**) PCoA plots of beta diversity for weighted and unweighted UniFrac distances. Ellipses represent the 95% confidence intervals for each group. Colors are assigned based on group allocation: red for the *Bf-688* group after supplementation, green for the *Bf-688* group before supplementation, blue for the placebo group after supplementation, and purple for the placebo group before supplementation. (**C**) Bar charts depict the average abundances of phylum, genus, and species-level ASVs across the four different samples studied: *Bf-688* group after supplementation, *Bf-688* group before supplementation, placebo group after supplementation, and placebo group before supplementation. “Other” refers to all species representing less than 10% abundance after the 10th most abundant species.

Initially, alpha diversity analysis, including the Simpson and Chao-1 indices, was conducted to examine differences in gut microbiota composition before and after treatment. Both the placebo and *Bf-688* groups exhibited a significant increase in microbial evenness after treatment, as depicted in Figure 4A. However, there were no significant differences in the number of rare microbial species (Chao-1 index) between the *Bf-688* and placebo groups (Figure 4A). Figure 4B reveals significant variations in beta diversity analysis between the two groups before and after the 12-week treatment period. To elucidate the microbial communities, we initially analyzed the dominant microbes at the phylum, genus, and species levels, as depicted in Figure 4C. Among the top ten phyla, notable differences in abundance were observed specifically in *Firmicutes* and *Bacteroidetes* before and after the intervention, and this was significant solely in the *Bf-688* group. Furthermore, when comparing the *Firmicutes* to *Bacteroidetes* (*F/B*) ratio, a significant increase was observed in the *F/B* ratio of the *Bf-688* group following the intervention, in contrast to the placebo group.

Further examination of the top ten genera and species did not reveal any significant alterations before and after the intervention, as depicted in Figure S1. To visually represent the significant differences in microbial communities between the placebo group (on the left) and the *Bf-688* group (on the right) before and after the intervention, a heatmap was utilized (Figure S2). For instance, in the placebo group, only five bacteria displayed significant changes in their richness before and after the intervention, and the corresponding bacteria in the *Bf-688* group were presented in the same heatmap. Notably, both groups exhibited similar trends based on significant differences in microbial communities before and after the intervention.

Given that all participants received consistent and appropriate treatment throughout the study and any potential influence attributable to the placebo was excluded in accordance with ethical principles governing human trials, we employed a Venn diagram analysis to delineate distinct and significant differences in gut microbiota before and after the intervention with *Bf-688* (Figure S3). This analysis allowed us to identify the specific gut bacteria that were truly affected by *Bf-688*. Among these, 20 gut bacteria displayed significant differences before and after the administration of *Bf-688*. Subsequently, we presented box plots to visually depict these differences individually (Figure S4).

**Figure 5:**
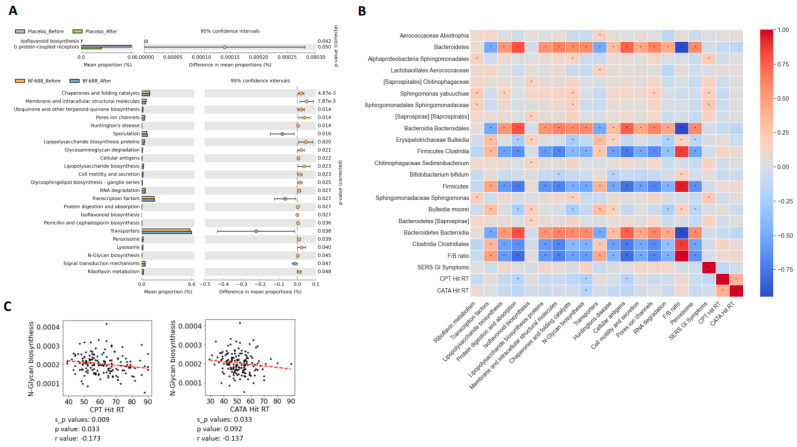
Correlations between CPT Hit reaction time (CPT Hit RT), CATA Hit reaction time (CATA Hit RT), and N-Glycan Biosynthesis In this extended error bar chart, we compare the predicted KEGG functional data based on four different samples: the *Bf-688* group before and after supplementation, and the placebo group before and after supplementation. Welch’s *t*-tests were applied, and only predicted functions with *p* < 0.05 between the two groups are displayed. (**A**) The left bar chart represents the mean proportions of each KEGG pathway, while the right dot plot illustrates the differences in mean proportions between the two compared sample groups, along with their respective *p*-values. We employed the PICRUSt to predict the potential mechanisms through which gut bacteria might be involved in these comparisons. (**B**) We present the correlations between microbial communities, pathways, and clinical parameters, including the *F/B* ratio, SERS GI symptoms, CPT Hit reaction time, and CATA Hit reaction time. * *p*-values (**C**) Significant correlations were observed between CPT Hit RT, CATA Hit RT, and N-Glycan Biosynthesis.

We extended our analysis by employing PICRUSt to predict potential mechanisms through which gut bacteria might be involved (Figure 5A). In order to uncover the specific pathways influenced by *Bf-688*, we scrutinized the distinct and significant differences in pathways before and after the intervention of *Bf-688* using a Venn diagram (Figure S5). This examination revealed six pathways that exhibited significant differences before and after the administration of *Bf-688*. These pathways encompassed Riboflavin metabolism, Transcription factors, Lipopolysaccharide biosynthesis, protein digestion and absorption, Lipopolysaccharide biosynthesis proteins, and N-Glycan biosynthesis. To illustrate the variations before and after the administration of *Bf-688* compared to the placebo group, these pathways were presented using box plots (Figure S6).

We explored correlations involving the *F/B* ratio, GI symptoms, CPT Hit RT, CATA Hit RT, and the microbial communities and pathways (Figure 5B). Notably, significant correlations were identified between N-Glycan biosynthesis and CPT Hit RT as well as CATA Hit RT (Figure 5C). Furthermore, N-Glycan biosynthesis exhibited negative correlations with *Firmicutes*, *Clostridia*, *Clostridiales*, and the *F/B* ratio, while it displayed positive correlations with *Bacteroidetes*, *Bacteroidia*, and *Bacteroidales* (Figure S7a). Additionally, CPT Hit RT showed a negative correlation with protein digestion and absorption. Moreover, protein digestion and absorption displayed negative correlations with the *F/B* ratio, *Firmicutes*, *Clostridia*, *Clostridiales*, *Bulleidia*, and *Bulleidia moorei*. Conversely, protein digestion and absorption exhibited positive correlations with *Bacteroidetes*, *Bacteroidia*, and *Bacteroidales* (Figure S7b).

## 4. Discussion

This study represents the inaugural randomized double-blind trial investigating the effects of *B. bifidum* (*Bf-688* strain) supplementation on clinical symptoms and the associated gut microbiome in individuals with ADHD. The primary focus of this study was on assessing GI symptoms and body weight changes in individuals undergoing MPH treatment. Although the results from this trial indicated that *Bf-688* did not lead to additional changes in body weight, the *Bf-688* group exhibited a superior outcome compared to the placebo group in terms of GI side effects by the study’s endpoint at week 12. Specific strains within the *Lactobacillus* and *Bifidobacterium* genera have demonstrated effectiveness in reducing body weight in individuals with overweight or obesity [64]. One review article highlighted the significant positive effects of *Bifidobacterium animalis* subsp. *lactis 420* (*B420*) on weight management and metabolic health through a complex network of signaling pathways, including those related to epithelial barrier function and the gut immune system. In the current study, we observed significant differences in the composition of *Firmicutes* and *Bacteroidetes* after the intervention in the *Bf-688* group. Moreover, there was a noteworthy increase in the *F/B* ratio in the *Bf-688* group, although no such increase was observed in the placebo group. An elevated *F/B* ratio has been associated with conditions like obesity and various diseases [65]. Despite these findings, *Bf-688* did not result in additional body weight gain effects in our study. In summary, our results suggest that *Bf-688* supplements may have the potential to alleviate GI symptoms by modulating gut microbiome dysbiosis in individuals undergoing MPH treatment.

Both parent-rated (SNAP-IV) and clinician-rated (ADHD-RS) assessments demonstrated significant reductions in both study groups over the 12-week period. However, the *Bf-688* group outperformed the placebo group in terms of reduced omission errors and improved response rates in visual attention (CPT) and auditory attention (CATA). Numerous strains of *B. bifidum* have been shown to have positive effects on various physical conditions. For example, *B. bifidum G9-1* (*BBG9-1*) has the potential to address dysbiosis, resulting in the modulation of organic acids and neurotransmitters like dopamine [36]. *Bifidobacterium* strains may contribute to phenylalanine production, which is involved in dopamine synthesis [31,32]. Importantly, disturbances in neurochemical mechanisms that affect the synthesis of monoamine neurotransmitters have been implicated in the pathophysiology of ADHD [66].

In comparison to the SNAP-IV and ADHD-RS scales, the CPT and CATA assessments provide relatively objective measures for evaluating neuropsychological function. Our previous research has indicated that CPT performance, rather than behavioral symptoms, tends to exhibit associations with biological markers like cytokines [55] or neuroendocrine markers [67]. We propose that *Bf-688* may indirectly influence catecholamine function in individuals with ADHD, resulting in improvements in visual and auditory attention. Furthermore, it has been suggested that the abundance of *Bifidobacterium* could potentially serve as a biomarker for ADHD due to its involvement in the dopamine neural reward system [32,68]. Our findings demonstrate that *Bifidobacterium bifidum* was indeed present at very low levels in these ADHD patients, and its abundance significantly increased following *Bf-688* supplementation, suggesting potential benefits for individuals with ADHD.

Our findings reveal meaningful correlations between CPT Hit RT, CATA Hit RT, and N-Glycan Biosynthesis, establishing a link between gut dysbiosis-induced N-glycosylation abnormalities and neurodevelopmental disorders like ADHD. N-glycosylation plays a crucial role in multicellular life, and its complete absence can be fatal during embryonic development [69]. Dysregulation of glycosylation is linked to a wide spectrum of diseases, including cancer, diabetes, cardiovascular disorders, congenital diseases, immunological disorders, and infectious diseases [70,71,72]. Variations in glycosylation have profound physiological significance because alterations in glycans can significantly impact the structure and function of glycoprotein polypeptide components [73]. Interestingly, prior research has shown increases in the glycan groups GP11 and DG7, along with a decrease in GP12, in the plasma of individuals with ADHD [74]. It is conceivable that differences in glycosylation efficiency could lead to changes in neural signaling and potentially contribute to the development of ADHD. Therefore, our results suggest that the beneficial effects of *Bf-688* on ADHD may be mediated through the reshaping of gut dysbiosis and the normalization of abnormal N-glycosylation processes.

Our study has several limitations. Firstly, although it was a randomized controlled trial, the placebo containing maltodextrin and corn starch could potentially have influenced the gut microbiome during the study period. Secondly, despite our efforts to control for dietary habits, lifestyle factors, and the use of antibiotics and anti-inflammatory drugs, other environmental factors may still have affected the gut microbiota. Thirdly, genetic variations could play a role in biological pleiotropy and the causal relationship between microbiota and psychiatric disorders [75]. The gut microbiome is a complex ecosystem with interdependent taxa involved in intricate interactions along with host genes and reaction pathways, some of which are related to neurotransmitters that play a role in ADHD neurocircuitry [76]. However, in our current study, we did not directly measure genetic variants, neurotransmitter levels, immune markers, or SCFA. Additional research is necessary to elucidate the molecular mechanisms that underlie the gut–brain axis and form the basis for modifying commensal microbiota or their functions as a potential therapeutic approach for ADHD [77,78]. Fourthly, the proportion of girls in the study was small, so caution is needed when making general recommendations. Additionally, because the study focused on children from Asia, further investigation is necessary to determine whether the results can be generalized to children from other continents. Lastly, the 12-week study period was short and might be insufficient to detect potential behavioral and neuropsychological functional changes in ADHD patients.

## 5. Conclusions

This study represents the first double-blind randomized trial to investigate the clinical and gut microbiome effects of *B. bifidum* (*Bf-688* strain) supplementation in children with ADHD undergoing pharmacotherapy. The results indicate that the probiotic *Bf-688* supplement was linked to improvements in visual and auditory attention in these ADHD children, possibly through its role in reshaping the composition of the gut microbiota and reducing N-Glycan biosynthesis. These findings suggest the potential benefits of probiotic supplementation for children with ADHD who are undergoing pharmacotherapy.

## Figures and Tables

**Table 1:** Characteristics at baseline among children with ADHD pharmacotherapy allocated into the probiotic (*Bf-688*) group (*n* = 51) and the placebo group (*n* = 51).

Variables	Probiotic Group (*n* = 51)	Placebo Group (*n* = 51)	χ or t	*p*-Value
Sex, *n* (%)			0.703 ^a^	0.402
Boys	42 (82.4)	45 (88.2)		
Girls	9 (17.6)	6 (11.8)		
Age, years	9.1 ± 1.8	9.1 ± 1.8	−0.117	0.97
Height, cm	133.0 ± 12.0	133.4 ± 10.1	−0.151	0.88
Body weight, kg	28.8 ± 7.7	29.6 ± 8.0	−0.494	0.622
Body mass index (kg/m^2^)	16.0 ± 2.1	16.4 ± 2.7	−0.722	0.472
Birth weight, g	3046.3 ± 483.0	3182.2 ± 519.3	−1.369	0.174
ADHD subtype, *n* (%)			0.043 ^a^	0.835
Inattentive	17 (33.3)	18 (35.3)		
Hyperactive or combined	34 (66.7)	33 (64.7)		
Comorbidity, *n* (%)				
Oppositional defiant disorder	4 (7.8)	2 (3.9)	0.708 ^a^	0.678
Tic disorders	0 (0)	2 (3.9)	2.040 ^a^	0.495
Methylphenidate dose (mg/day)	22.1 ± 11.7	23.4 ± 10.0	−0.576	0.566
MPH formulation, *n* (%)			0.050	1.000
Short-acting MPH	14 (27.5)	13 (25.5)		
Long-acting MPH	37 (72.5)	38 (74.5)		
Barkley’s Side Effects Rating Scale				
Total score	14.8 ± 12.7	13.7 ± 9.4	0.487	0.628
Gastrointestinal symptoms	3.8 ± 3.5	4.0 ± 3.3	−0.174	0.862
SNAP-IV scores				
Inattention	14.7 ± 5.6	15.3 ± 6.2	−0.458	0.648
Hyperactivity/impulsivity	12.8 ± 6.1	12.7 ± 7.0	0.137	0.891
ADHD-RS scores				
Inattention	11.7 ± 5.4	11.5 ± 6.7	0.197	0.844
Hyperactivity/impulsivity	13.9 ± 4.9	13.3 ± 7.1	0.462	0.645
CPT				
Detectability (d’)	50.1 ± 9.7	48.6 ± 10.7	0.756	0.451
Omissions	53.0 ± 12.6	53.5 ± 12.2	−0.135	0.893
Commissions	47.2 ± 8.9	46.1 ± 8.6	0.633	0.528
Hit reaction time (RT)	62.7 ± 10.5	61.6 ± 13.2	0.481	0.632
CATA				
Detectability (d’)	51.6 ± 9.4	50.3 ± 10.0	0.663	0.509
Omissions	49.2 ± 8.6	48.7 ± 7.6	0.328	0.743
Commissions	52.1 ± 11.6	51.8 ± 12.4	0.14	0.889
Hit reaction time (RT)	53.7 ± 10.6	51.3 ± 9.7	1.187	0.238

Notes: Data are expressed as mean ± SD or *n* (%); ^a^ Pearson Chi-square; CPT, Conners’ Continuous Performance Test; CATA, Conners’ Continuous Auditory Test of Attention; Hit reaction time (RT); ODD, oppositional defiant disorder; SNAP-IV, the Swanson, Nolan, and Pelham Rating Scale; ADHD-RS, ADHD rating scale; SERS, Barkley’s Side Effects Rating Scale; SERS gastrointestinal symptoms, severity of stomachache, and loss of appetite.

## Data Availability

The data presented in this study can be obtained upon request from the corresponding author due to ethical.

## Supplementary Materials

The following supporting information can be downloaded at https://www.mdpi.com/article/10.3390/nu16142260/s1, Figure S1. Changes in Microbial Phyla, Genera, and Species Before and After Intervention. Figure S2. Heatmap Visualization of Significant Differences in Microbial Communities. Figure S3. Venn Diagram Analysis of Gut Microbiota Changes. Figure S4. Changes in the Abundances of Gut Bacteria with *Bf-688* Administration. Figure S5. Venn Diagram Illustrating Unique Significant Pathway Differences Before and After *Bf-688* Intervention. Figure S6. Box Plots Illustrating Changes in Pathways Before and After *Bf-688* Administration. Figure S7. Correlation Analysis of N-Glycan Biosynthesis and Protein Digestion and Absorption with Gut Microbiota Taxa.
