# Supplementary material for: Add-On Bifidobacterium Bifidum Supplement in Children with Attention-Deficit/Hyperactivity Disorder: A 12-Week Randomized Double-Blind Placebo-Controlled Clinical Trial
Source: Nutrients. 2024 Jul 13;16(14):2260. doi: 10.3390/nu16142260 (PMC11279422; doi:10.3390/nu16142260)
Supplement: Supplementary file 1 [file nutrients-16-02260-s001.zip › nutrients-3091004-supplementary.pdf]

Supplementary Materials

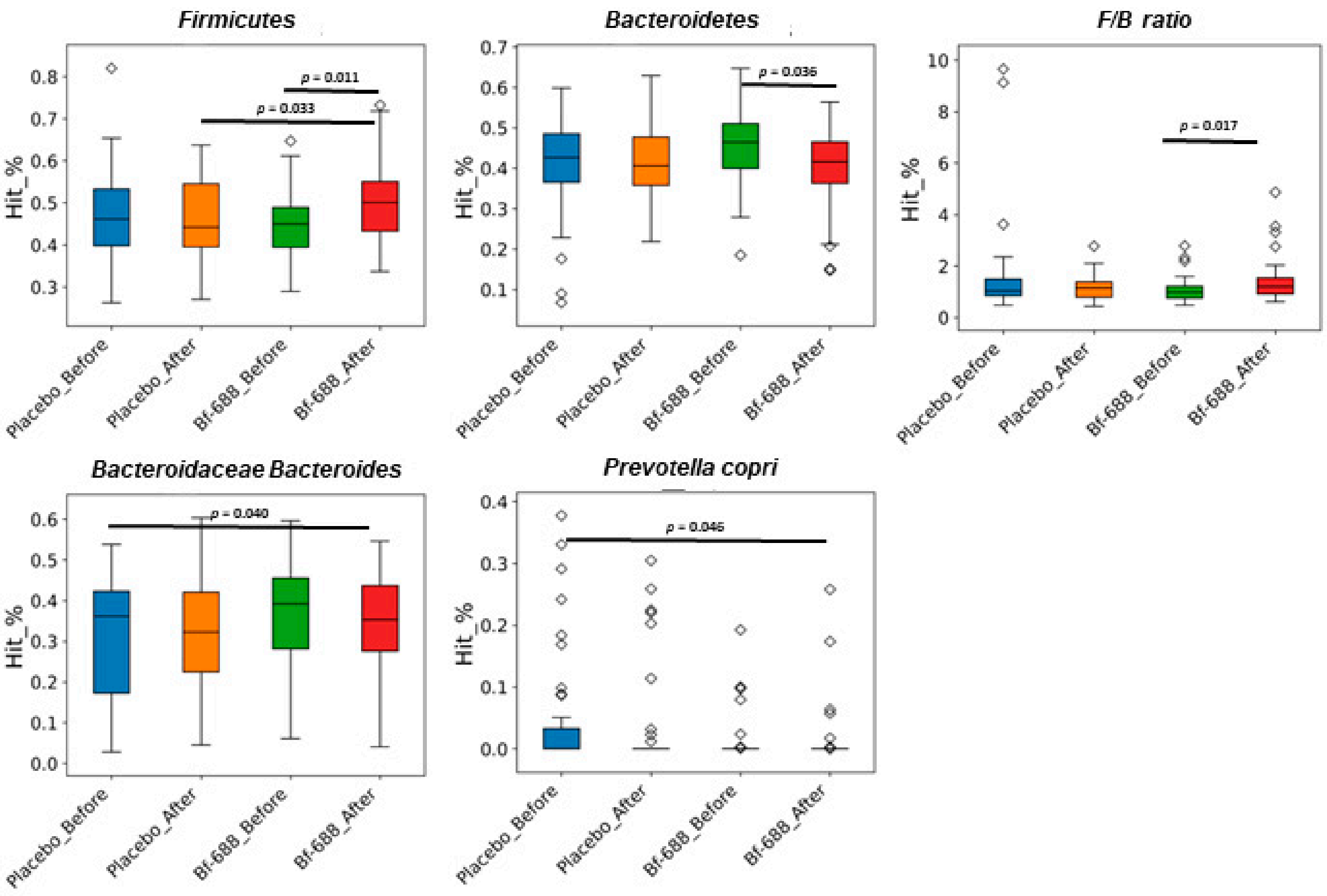

Figure S1. Changes in Microbial Phyla, Genera, and Species Before and After Intervention This figure illustrates the alterations in the composition of the top 10 phyla, genera, and species within the gut microbiota in response to the intervention.

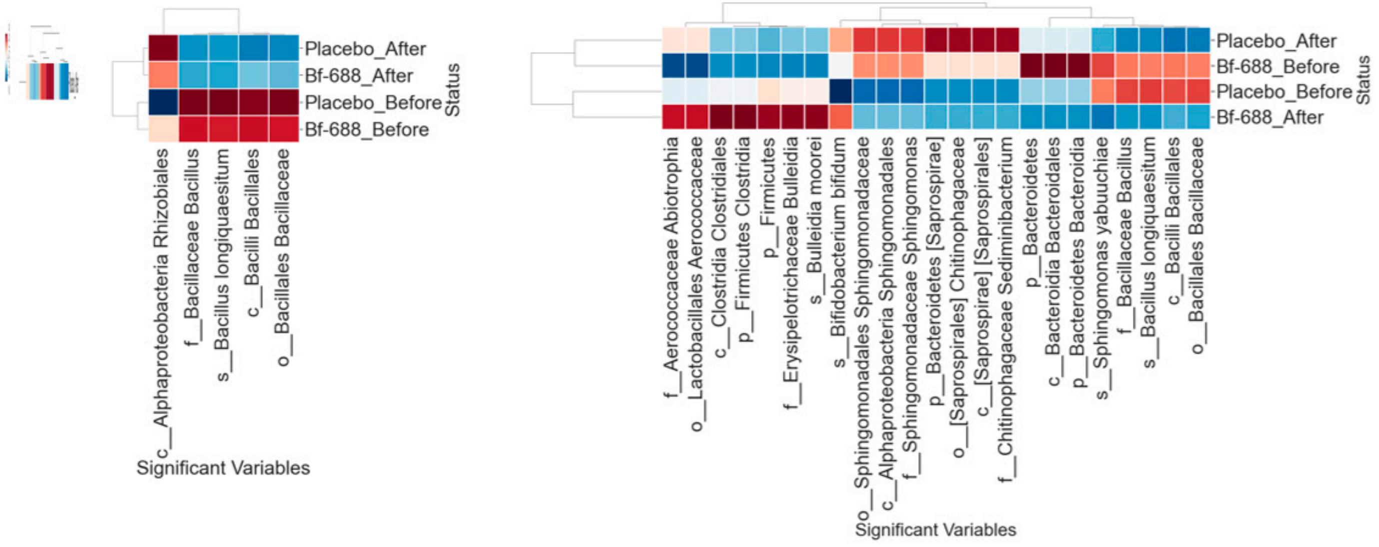

Figure S2. Heatmap Visualization of Significant Differences in Microbial Communities. A heatmap is employed to illustrate all significant differences ($p < 0.05$, Wilcoxon rank-sum test) in microbial communities observed between Placebo (**left**) and Bf-688 group (**right**) before and after the intervention. We utilized a heatmap to illustrate all statistically significant differences (Wilcoxon rank sum test $p < 0.05$) in microbial communities between the Placebo (**left**) and Bf-688 group (**right**) before and after the intervention.

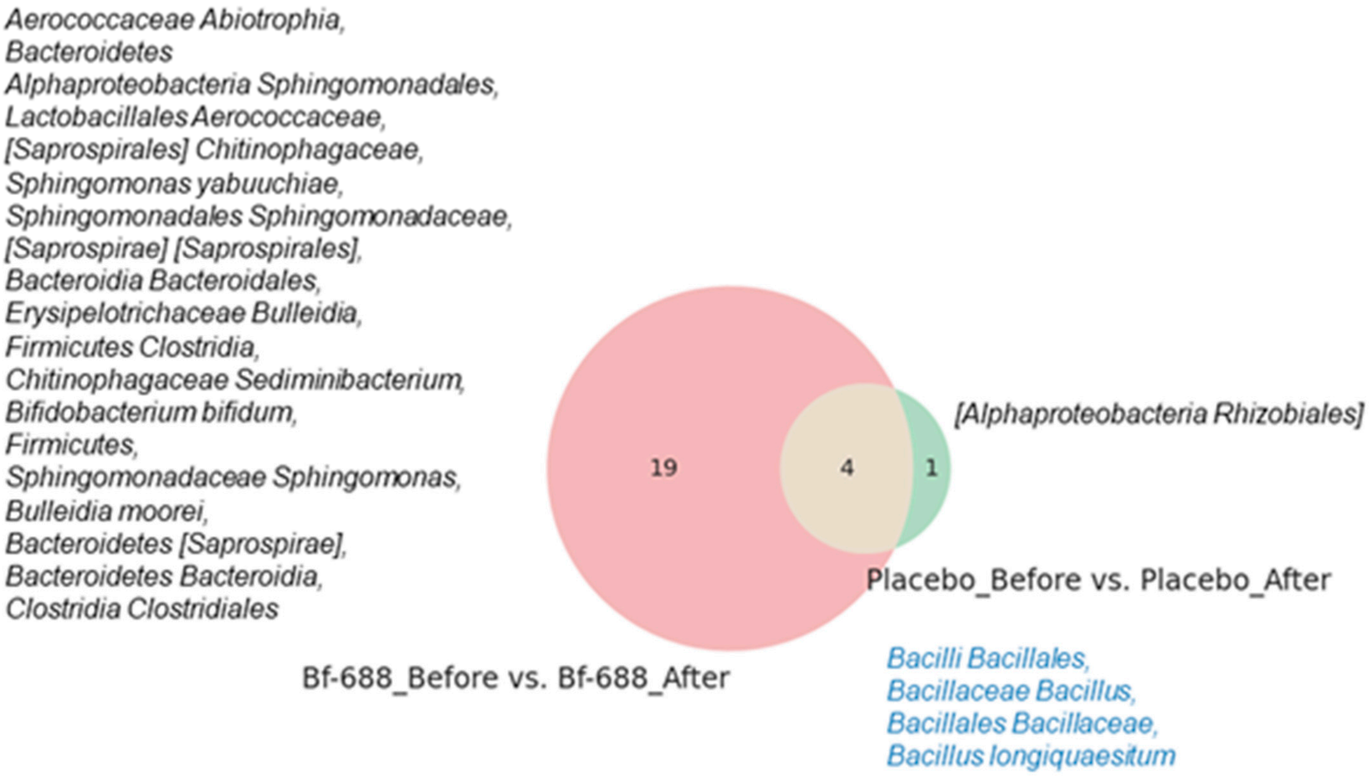

Figure S3. Venn Diagram Analysis of Gut Microbiota Changes This figure illustrates the results of a Venn diagram analysis conducted to investigate the distinct and significant differences in gut microbiota composition before and after the intervention with Bf-688. As all participants received continuous and appropriate treatment throughout the study, potential influences caused by the placebo were excluded in accordance with ethical principles governing human trials. The Venn diagram provides insights into the specific gut bacteria that were genuinely impacted by the administration of *Bf-688*.

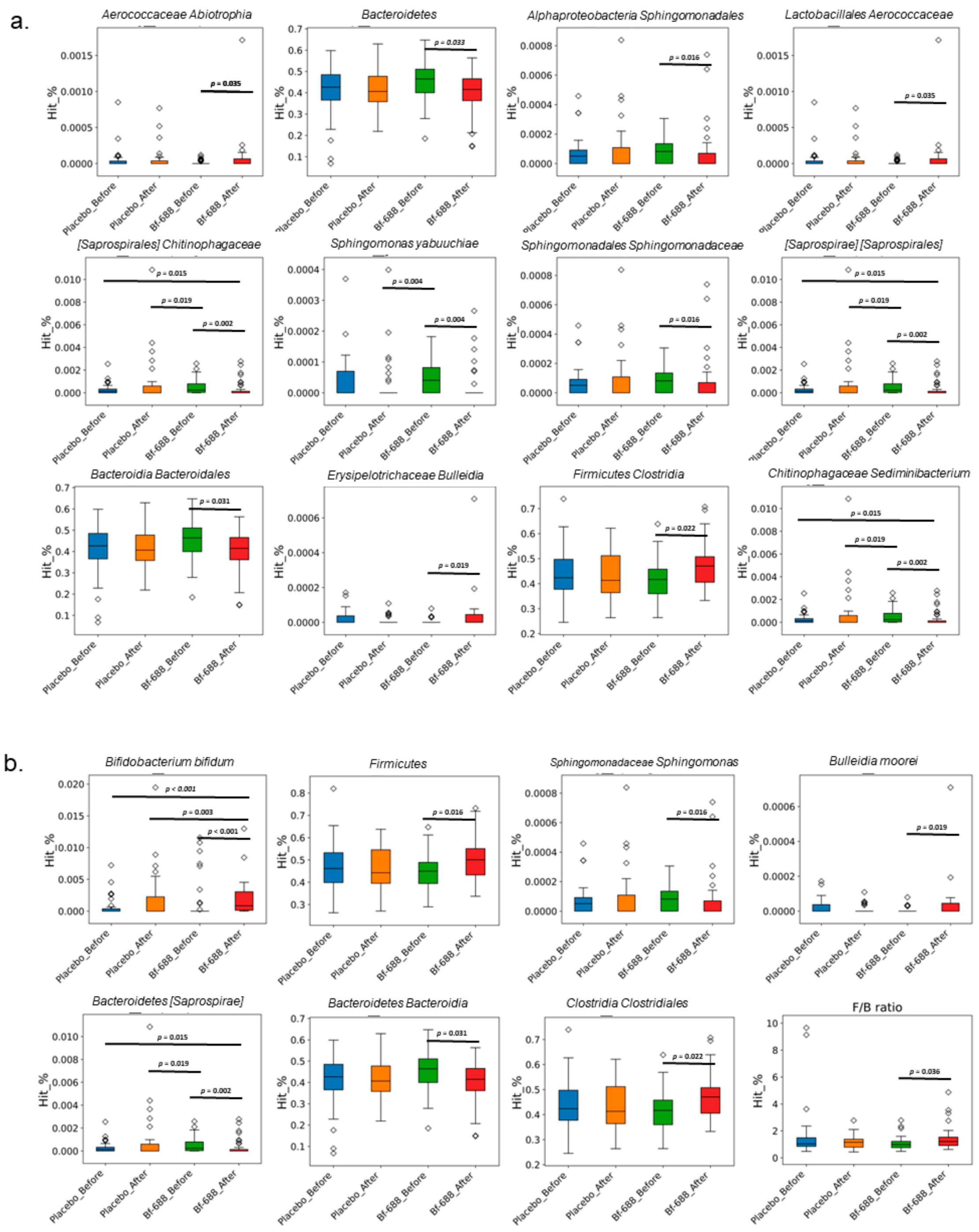

Figure S4. Changes in the Abundances of Gut Bacteria with *Bf-688* Administration This figure presents the alterations in the abundances of 20 specific gut bacteria before and after the administration of *Bf-688*. Boxplots are employed to visually represent these significant differences, highlighting the impact of *Bf-688* on the gut microbiota composition.

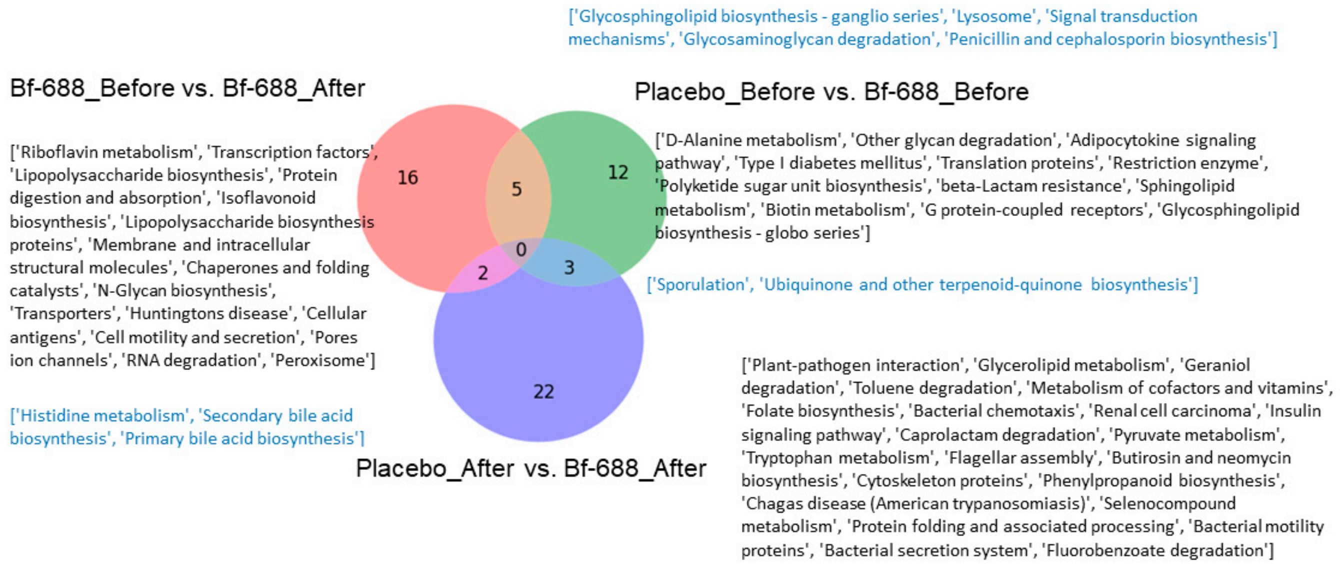

Figure S5. Venn Diagram Illustrating Unique Significant Pathway Differences Before and After *Bf-688* Intervention.

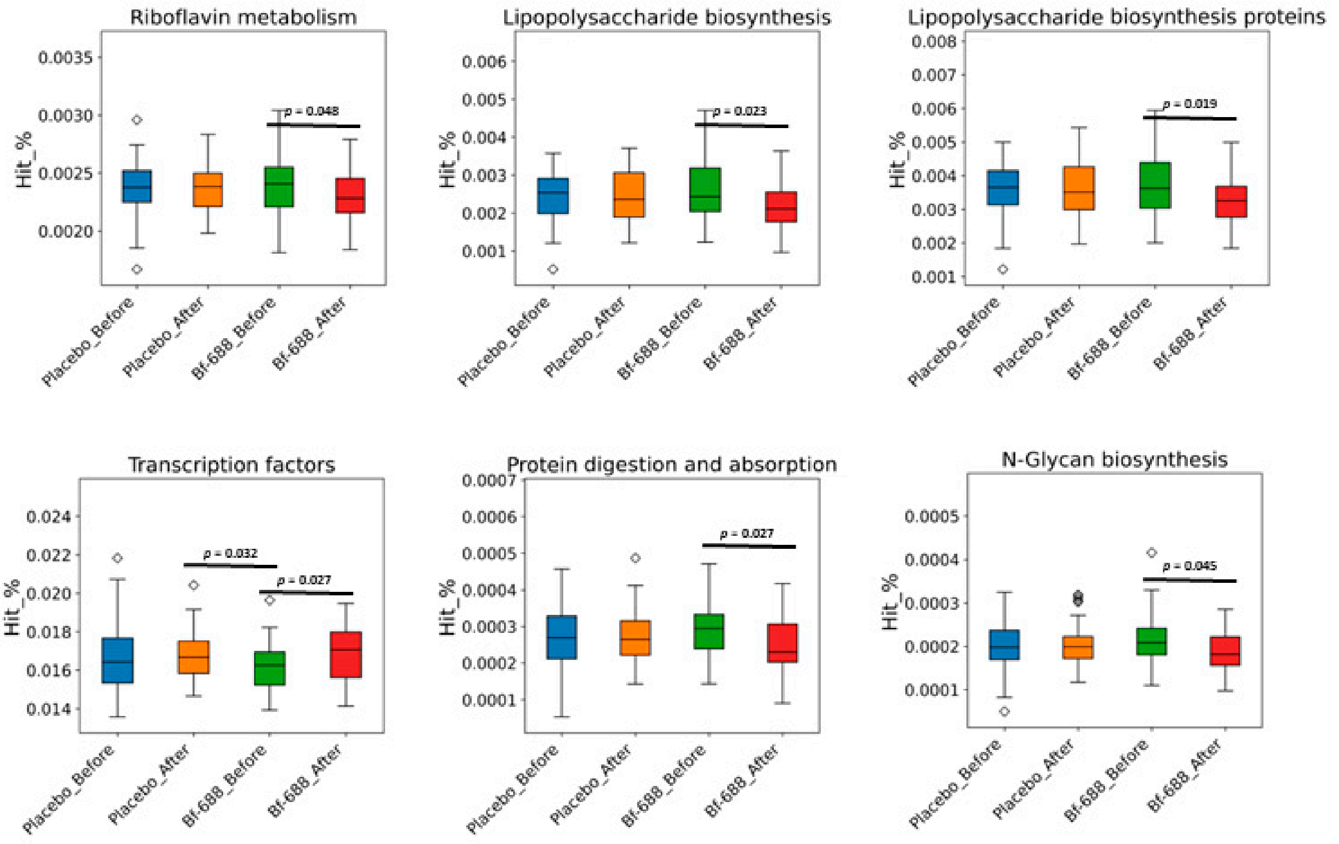

Figure S6. Box Plots Illustrating Changes in Pathways Before and After *Bf-688* Administration This figure displays box plots representing the variations in six pathways (i.e., Riboflavin metabolism, Transcription factors, Lipopolysaccharide biosynthesis, Protein digestion and absorption, Lipopolysaccharide biosynthesis proteins, and N-Glycan biosynthesis) before and after the administration of *Bf-688*. The box plots provide a visual representation of the changes in pathway activity, allowing for a comparative analysis of pathway dynamics between two time points.

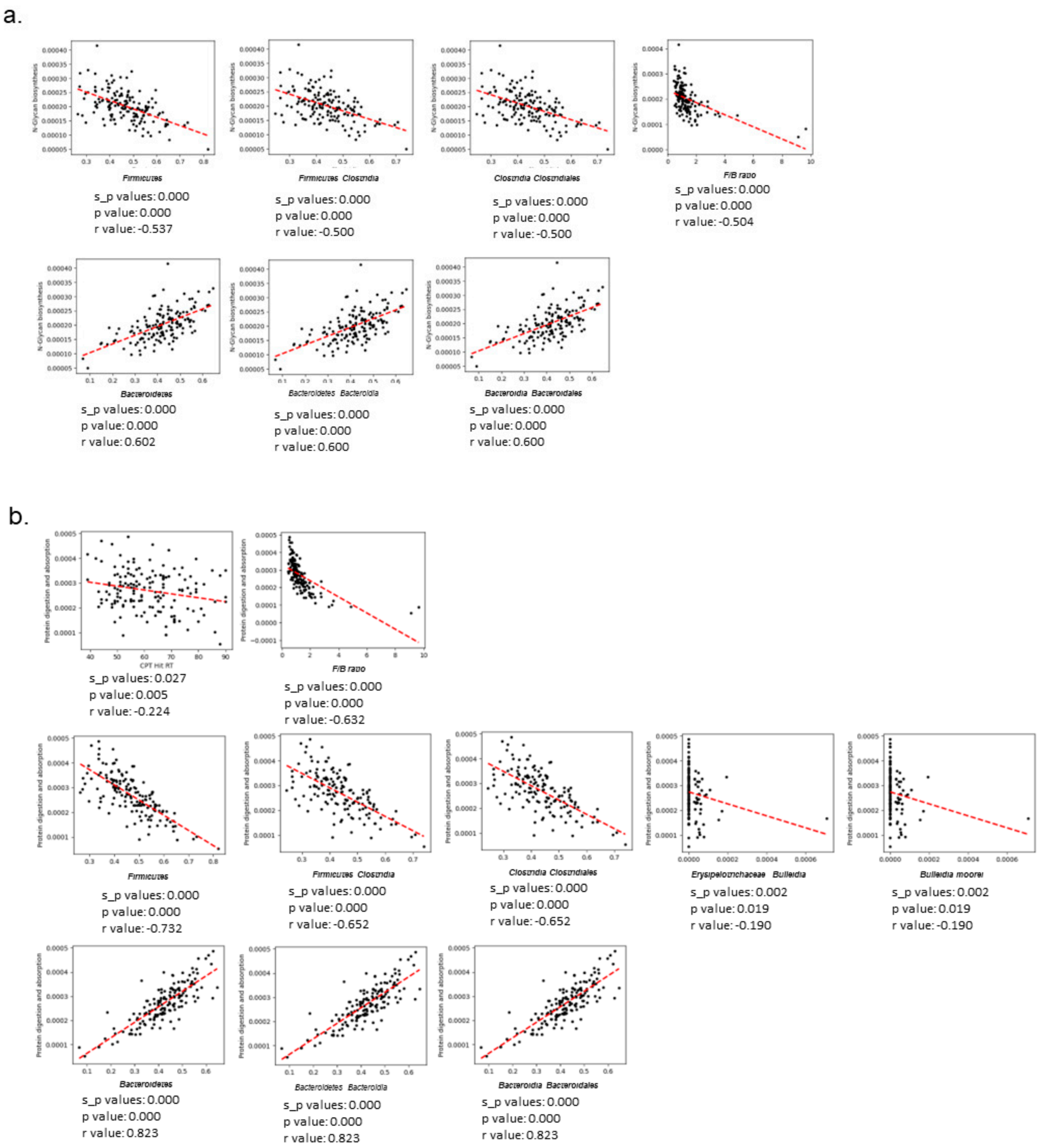

Figure S7. Correlation Analysis of N-Glycan Biosynthesis and Protein Digestion and Absorption with Gut Microbiota Taxa.
